# Assessment of a multisite standardized biospecimen collection protocol for immune phenotyping in neurodevelopmental disorders

**DOI:** 10.1038/s41598-023-33380-z

**Published:** 2023-04-28

**Authors:** Shane Cleary, Grace Teskey, Craig Mathews, Russell J. Sachachar, Robert Nicolson, Rosanna Weksberg, Evdokia Anagnostou, Dawn M. E. Bowdish, Jane A. Foster

**Affiliations:** 1 Department of Psychiatry and Behavioural Neurosciences, McMaster University, Hamilton, ON Canada; 2 The Research Institute at St. Joe’s, Hamilton, ON Canada; 3 Department of Medicine, McMaster University, Hamilton, ON Canada; 4 Department of Psychiatry, University of Toronto, The Hospital for Sick Children, Toronto, ON Canada; 5 Lawson Health Research Institute and Western University, London, ON Canada; 6 Division of Clinical and Metabolic Genetics and Genetics and Genome Biology Program, The Hospital for Sick Children, Toronto, ON Canada; 7 Departments of Pediatrics, University of Toronto, Toronto, ON Canada; 8 Department of Molecular Genetics, University of Toronto, Toronto, ON Canada; 9 Institiute of Medical Sciences, University of Toronto, Toronto, ON Canada; 10 Bloorview Research Institute, Holland Bloorview Kids Rehabilitation Hospital, Toronto, ON Canada; 11 McMaster Immunology Research Centre, McMaster University, Hamilton, ON Canada

**Keywords:** ADHD, Autism spectrum disorders, Immunology

## Abstract

Multisite collection and preservation of peripheral blood mononuclear cells (PBMCs) for centralized analysis is an indispensable strategy for large cohort immune phenotyping studies. However, the absence of cross-site standardized protocols introduces unnecessary sample variance. Here we describe the protocol implemented by the Province of Ontario Neurodevelopmental Disorders (POND) Network's immune platform for the multisite collection, processing, and cryopreservation of PBMCs. We outline quality control standards and evaluate the performance of our PBMC processing and storage protocol. We also describe the Child Immune History Questionnaire results, an assessment tool evaluating pre-existing immune conditions in children with neurodevelopmental disorders (NDDs). Cell viability was assessed in samples from 178 participants based on strict quality control criteria. Overall, 83.1% of samples passed quality control standards. Samples collected and processed at the same site had higher quality control pass rates than samples that were collected and subsequently shipped to another site for processing. We investigated if freezer time impacted sample viability and found no difference in mean freezer time between samples that passed and failed quality control. The Child Immune History Questionnaire had a response rate of 87.1%. The described protocol produces viable samples that may be used in future immune phenotyping experiments.

## Introduction

Neurodevelopmental disorders (NDDs) are a diverse group of conditions defined by communication, behavioural, and cognitive deficits that result from atypical brain development^1–3^. NDDs include but are not limited to Autism Spectrum Disorder (ASD), Attention-Deficit/Hyperactivity Disorder (ADHD), Obsessive–Compulsive Disorder (OCD), Intellectual Disability (ID), Rett syndrome, Down syndrome, and other genetic disorders that influence neurodevelopment. The pathophysiology of NDDs is multifactorial, with studies reporting multiple genetic and environmental risk factors^4–8^. Furthermore, the clinical presentation of NDDs is heterogeneous, with shared symptoms across disorders and symptom severity varying between and within disorders^1–3^. Understanding the biological basis of this heterogeneity may aid in extending the current diagnostic criteria and provide novel biomarker-based approaches to guiding treatment and research.

There is evidence that the immune response contributes to the development and progression of NDDs. Increased levels of pro-inflammatory markers in the brain and periphery^9–11^, as well as differences in surface receptors, function, and counts of monocytes^12–15^ and lymphocytes^16–19^, have been reported in NDDs compared to typically developing (TD) individuals. Hyperactivity and deficits in social interactions and communication correlate with higher levels of inflammation in people with NDDs^20–23^, suggesting that inflammation and dysregulated immune responses may contribute to the heterogeneity of symptoms seen in NDDs. Stratifying individuals based on immune phenotype and identifying biomarkers specific to behavioural outcomes may be an effective option to precision health approaches for NDDs.

Studies on the immune system’s role in the etiology or symptomology of NDDs have been limited due to small sample sizes, unstandardized approaches to handling biospecimens, and inconsistencies in the types of cells and cellular markers analyzed. The immune research platform of the Province of Ontario Neurodevelopmental Disorder (POND) Network aims to conduct comprehensive immunophenotyping in a large cohort of people with different NDDs from multiple sites across the province. Flow cytometry is the tool of choice to analyze several cellular parameters; however, conducting multisite immunophenotyping is challenging. Studies have demonstrated that multiple factors, including equipment types, calibration, gating, and analysis, contribute to an increase in data variation when using flow cytometry across multiple sites^24–27^. Cryopreservation is commonly used to allow for centralized investigation of peripheral blood mononuclear cells (PBMCs), ameliorating the confounding factors present in multisite analyses^24–26^. Studies of long-term cryopreservation have shown that cell viability decreases by less than 1% every year that they are frozen, indicating minimal cell loss over time^28^. However, it has also been shown that cryopreservation may alter the counts of monocyte and lymphocyte populations and affect the expression of several surface receptors compared to fresh samples^29–31^. The differences in cell counts and surface receptor expression appear consistent across the literature, allowing for their consideration during experimental design^29–31^. These studies demonstrate the feasibility of using cryopreserved PBMCs to facilitate a centralized analysis approach for samples collected at multiple sites. This paper presents a standardized approach used to collect and bank biospecimens for immunophenotyping and assesses the viability of cryopreservation for centralized analysis in a multisite study of TD individuals and individuals with NDDs.

## Results

Banked biospecimens from 178 participants were analyzed. The participants' demographics are provided in Table 1. 82.6% of the participants had NDD (n = 147), with ASD making up 71.4% of the NDD diagnoses (n = 105). 81.5% of the participants were male, and 18.5% were female, which reflects the sex differences in NDD prevalence. The Child Immune History Questionnaire was available from 87.1% of participants (n = 155). Response and completion rates by diagnosis and questionnaire section can be found in Table 1. The "Infection" section of the questionnaire had a significantly lower response rate in the NDD group than in the TD group (*p* < 0.01). The proportion of participants who reported having an autoimmune disease or allergy did not differ between TD and NDD (*p* = 0.15). There was a higher proportion of people who reported having a “significant” infection in the NDD group compared to TD (*p* = 0.02). There was also a significantly higher proportion of individuals in the NDD group who reported a history of immunosuppressive medication use (*p* < 0.01).

**Table 1:** Demographic overview of participants & child immune history questionnaire responses.

	TD		NDD		*p*
	Males	Females	Males	Females	
*n*	17	14	128	19	
Median age (IQR)	13.0 (3.5)	14.0 (6.3)	11.5 (6.8)	10.0 (9)	< 0.01
Questionnaire response rate (%)	100	92.8	85.9	78.9	0.13
Complete questionnaires by section (%)					
Autoimmune/allergy^a^	100	100	98.1	93.3	0.99
Infection^b^	100	100	45.8	73.3	< 0.01
Medication^c^	100	92.3	87.1	86.6	0.2
Presence of immune condition (% of total)					
Autoimmune/allergy^a^	58.8	53.8	39.8	42.9	0.15
Infection^b^	70.6	61.5	88	100	0.02
Medication^c^	17.6	0	35.8	38.4	< 0.01

IQR—interquartile range.

^a^Autoimmune disease or allergy: allergy, asthma, allergic rhinitis, food allergy, autoimmune cytopenia, other autoimmune disease.

^b^Childhood Infections: Oral thrush in first year of life, any ear infections in past 3 years, nail fungus, skin warts, chicken pox, other self-described “significant” infection that required antibiotics use.

^c^Immunosuppressive Medication: Immunosuppressants, oral/IV steroid use.

### Viability of banked PBMCs

**Figure 1:**
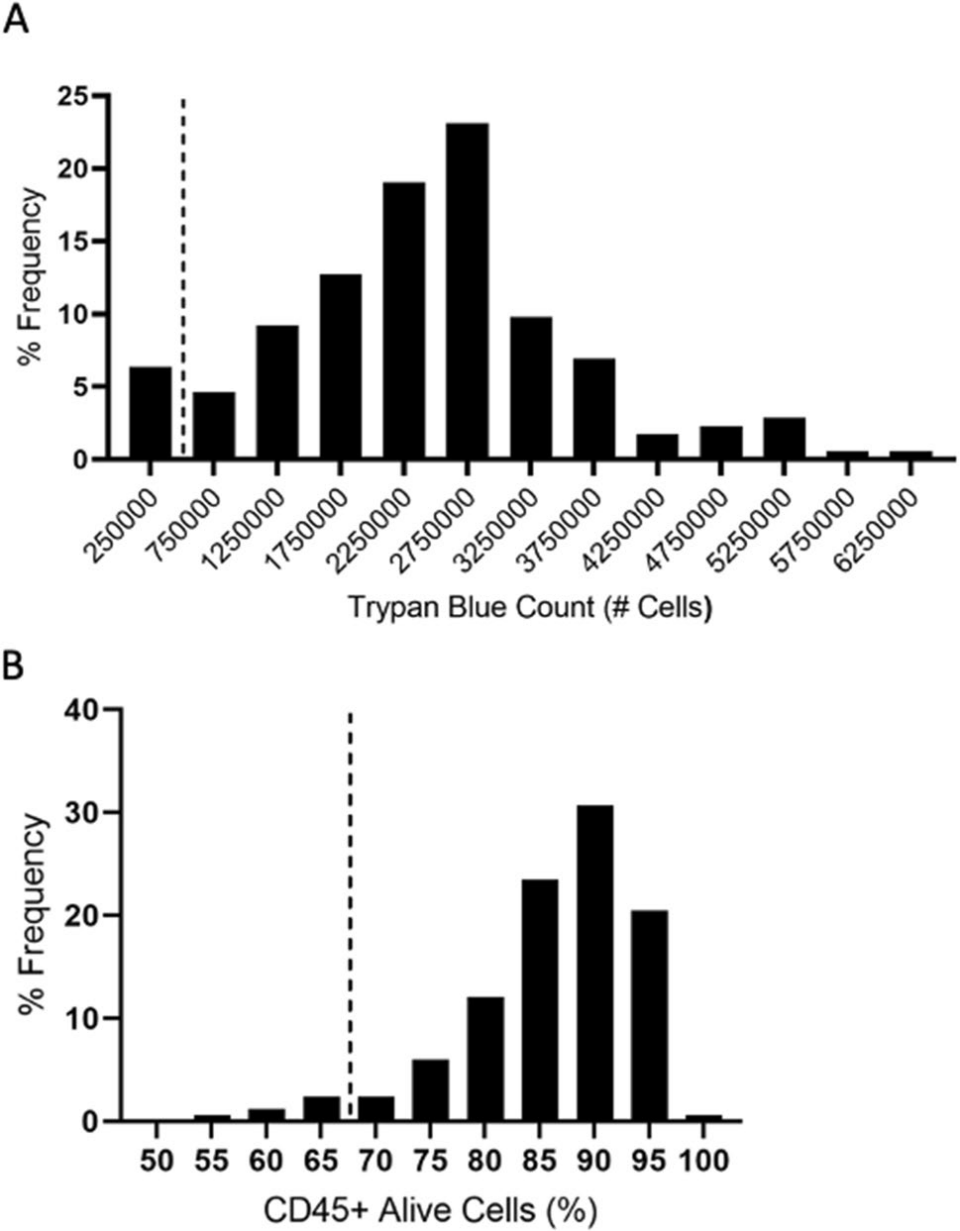
Quality control data. (**A**) Frequency distribution of Trypan Blue counts in thawed samples. The percent frequency of post-thaw sample PBMC counts measured using the Trypan Blue exclusion test. Samples containing more than 500,000 cells, indicated by the dotted line, proceeded to quality control phase two. (**B**) Frequency distribution of alive CD45+ Cells in thawed samples. The percent frequency of CD45+ cells that were negative for the Zombie Red viability stain as a percent of CD45+ cells in the sample. Samples with a survival rate of 70%, indicated by the dotted line, were considered viable for future use.

The viability of thawed PBMCs was determined using two different viability assessment methods. The Trypan Blue exclusion test was conducted on 178 PBMC samples, with 93.8% (n = 167) passing. These samples proceeded to a viability assessment using flow cytometry. A frequency distribution of Trypan Blue counts can be found in Fig. 1a. Of the 167 PBMC samples that proceeded to flow cytometry analysis, 88.7% (n = 148) passed the flow cytometry quality control criteria. A frequency distribution of the % alive CD45+ cells can be found in Fig. 1b. The overall quality control pass rate of cryopreserved PBMCs was 83.1% (Table 2).

**Table 2:** Quality control overview of cryopreserved PBMCS.

178 Samples analyzed	FACS				*p*
	Samples passed QC		Samples failed QC		
Quality control status	Total passed	148	Total failed	30	
			TB count	11	
			FACS viability	16	
			Granulocyte contamination	3	
Mean freezer time ± SE	Days	273.2 ± 10.4	Days	349.7 ± 34.5	0.11
	Months	9.0 ± 0.3	Months	11.5 ± 1.1	0.11

The viability of banked PBMCs by site is shown in Table 3. Early in sample collection, the transportation method from Site 1 to the processing site was changed from samples being transported on ice, to being transported at room temperature. This change led to an 32.1% increase in sample viability. Fisher’s exact test showed that samples processed on site had a significantly higher proportion of quality control passes than sites where samples were transported prior to processing (*p* < 0.01).

**Table 3:** Quality control pass/fail rate by site.

	% Passed	% Failed
Site		
Site 1—Pre^a^	42.9	57.1
Site 1—Post^b^	75.0	25.0
Site 2	74.4	25.6
Site 3	91.9	8.1
Site 4	96.8	3.2
Processing location		
On site	95.0	5.0
Transported	74.6	25.4

^a^Samples transported on ice prior to protocol change.

^b^Samples transported at room temperature post protocol change.

The mean time (± SE) spent in the freezer before the viability assessment was 286.1 ± 10.6 days. Samples that passed quality control spent 276.0 ± 11.1 days frozen, whereas samples that failed quality control spent 321.0 ± 27.3 days frozen. The difference in the number of days and months spent in the freezer between samples that passed or failed quality control was not significant (*p* = 0.11).Complete blood count (CBC) reports detailing absolute counts of lymphocytes, monocytes, neutrophils, eosinophils, and basophils were available for 62.9% of participants (n = 112). Of the participants with CBC reports, 89 passed and 23 failed quality control criteria for the Trypan Blue counts and % alive CD45+ cells. The profile of leukocytes available on the CBC reports were examined and showed that there were no differences in absolute counts of leukocytes (prior to banking) between the samples that passed and failed quality control (Supplementary Fig. 1).

## Discussion

This report provides an initial assessment of the viability and quality of the biospecimen collection protocol for the POND immune research platform and an analysis of participant immune-related medical history. The biospecimen collection protocol was designed to address the challenges associated with the multisite collection of PBMCs. Additionally, centralized storage and analysis are more cost-effective as less equipment and personnel are required at the collection sites. The Child Immune History Questionnaire was implemented to collect data on conditions that may influence immune status, allowing for their consideration when conducting immune phenotyping.

Other studies have examined the effects of long-term cryopreservation on PBMCs^28,29^. A study assessing the viability of PBMCs cryopreserved from multiple sites for up to 12 years found that the median viability was 90%, with a minimum viability of 82%^28^. Additionally, a study assessing cryopreservation of immune cells in whole blood found viability of 58 ± 3% after four years; however, most of the loss was attributed to neutrophils^29^. The authors reported a monocyte viability of 68 ± 8% and a lymphocyte viability of > 90%^29^. It is important to note that in our described protocol granulocytes are isolated and stored separately from other PBMC populations. The ranges provided by both studies are comparable to the overall PBMC viability of 83.1% in the present study, determined by the described quality control standards.

Site-wise comparisons indicate that immediate processing provides better sample viability than transporting samples from a collection site to a processing site. Additionally, transportation conditions appear to be a significant factor in sample viability. Changing the ttransportation protocol from on ice to room temperature greatly increased sample viability of site 1.

To date, attempts to characterize a comprehensive immune phenotype of those with NDDs are limited^16,18^. These studies used flow cytometry to analyze PBMC populations in children with ASD and TD controls, with one study using fresh blood samples and the other using cryopreserved PBMCs^16,18^. While both studies found immune cell population differences between ASD and TD, their findings are limited to young children between 5 and 7 years old^16,18^. Expanding the age ranges is important. The POND immunophenotyping platform recruits individuals between 1 and 23 years old with various NDDs.

Sample processing and cryopreservation present two important limitations. The first limitation is due to the processing involved in sample preparation. As the volume of blood collected varies and cells are lost with each wash cycle, absolute counts of cell populations are not comparable to counts obtained using fresh blood samples. Therefore, PBMC populations were reported as %CD45+ cells or standardized to cells per million. Additionally, it has been reported that cryopreserved PBMCs yield different cell counts of monocyte and lymphocyte populations when compared to fresh blood^28,29^. However, these variations in monocyte and lymphocyte counts appear to be consistent across the literature and are therefore an inherent limitation of cryopreservation which needs to be considered during study design^29,30^. The collection of CBC reports in conjunction with PBMCs will be used in future studies to further assess potential differences between cryopreserved counts and fresh blood counts. The second identified limitation is that cryopreservation has been shown to reduce the mean fluorescent intensity of specific cell markers on monocytes, lymphocytes, and granulocytes, making less abundant markers more difficult to detect^29^. Furthermore, cryopreservation reduces the reliability of surface markers of activation CD45RA and CD62L despite the cells remaining viable^32^. The surface markers with reduced reliability appear to be consistent across the literature^31,32^. If unreliable surface markers are of interest, flow cytometry should be conducted on fresh blood samples instead of on cryopreserved samples. This limits the ability to conduct a multisite analysis of these markers, and therefore should be addressed during study design. In summary, the POND Network immune platform has designed and implemented a protocol for multisite sample collection and single site storage and provided an assessment of the viability and quality of samples procured. The protocol allows for the collection of biospecimens from individuals with a variety of NDDs across the Province of Ontario and provides a framework for reliable immunophenotyping. This study will allow for an extensive analysis of immunophenotyping in NDDs and provide insight into immune biomarkers linked to the clinical presentation of NDDs.

## Methods

### Participants

Participants were recruited through the POND Network (pond-network.ca) at 5 sites: Holland Bloorview Kids Rehabilitation Hospital, Toronto; The Hospital for Sick Children, Toronto; Queens University, Kingston; Lawson Health Research Institute, London; and McMaster Children's Hospital, Hamilton. Primary caregivers and participants provided either written informed consent or assent after a complete description of the study was provided in accordance with the Tri Council Policy Statement, the Declaration of Helsinki, and the International Council for Harmonization of Technical Requirements for Pharmaceuticals for Human Use (ICH)–Good Clinical Practice (GCP) guidelines and institutional policy. Approval for collection and sample banking were obtained from the research ethics boards of each institution including Holland Bloorview Research Ethics Board, Sick Kids Research Ethics Board, General Research Ethics Board at Queen’s University, Western University’s Health Sciences Ethics Board, and Hamilton Integrated Research Ethics Board.

A sub-cohort of children and young adults with NDDs and TD participants that contributed immune samples between January 2017 and July 2019 were enrolled. Eligible participants were between the ages of 1 and 23 years with a diagnosis including ASD, ADHD, OCD, and ID, in addition to TD controls. Diagnoses were confirmed using standardized assessment tools. ASD was confirmed using the Autism Diagnostic Observation Schedule (ADOS) and the Autism Diagnostic Interview-Revised (ADI-R), ADHD was confirmed using the Parent Interview for Child Symptoms (PICS), and OCD was confirmed using the Children's Yale-Brown Obsessive–Compulsive Scale (CY-BOCS)^33–36^. Exclusion criteria for the immune platform were as follows: fever greater than 38 °C in the preceding 3 days, vaccination in the preceding 7 days, oral or intravenous steroids in the preceding 14 days, oral or intravenous antibiotics in the preceding 21 days, chemotherapy, intravenous immunoglobulins of monoclonal antibodies in the preceding 12 months and known primary or secondary immunodeficiency.

### Immune metrics

The immune medical history of participants was assessed using the Child Immune History Questionnaire (Supplemental File 1) to collect information about the participants' immune status. The questions were grouped into three sections for analysis: autoimmune/allergy, childhood infection, and medication. Autoimmune/allergy-related questions ascertained if the participant had environmental, food, or other allergies, asthma, autoimmune cytopenia, or other autoimmune diseases. The childhood infection questions collected information about the participant's history of infections, including oral thrush, ear infections, skin warts, chickenpox, or other self-described “significant” infections that required antibiotic use. Lastly, the medication section questions determined if the participant had previously used immunosuppressants or oral/IV steroids. Sections were considered incomplete if at least one question was left blank. Participants with incomplete responses were removed using case-wise deletion.

### Biospecimen collection

Venous blood was drawn from participants: (1) in a 10 mL heparinized blood tube for the isolation of PBMCs, granulocytes and plasma (367,874, Becton, Dickinson and Company, Mississauga, ON); (2) in two 5 mL silica-coated serum tubes for the isolation of serum (366,434 G, Becton, Dickinson and Company, Mississauga, ON); (3) 4 mL EDTA treated tube for complete blood counts (367,844, Becton, Dickinson and Company, Mississauga, ON). Anonymized samples (1 and 2) were transported from the blood collection site to the nearest processing lab, where biospecimens were cryopreserved. The EDTA samples (3) were transported to 3rd party medical labs for CBC analysis. The date and time of the blood draw were recorded. Biospecimen samples were processed with minimal delay after collection. Samples collected in Hamilton and London areas were immediately transported to the processing lab at McMaster University. Samples collected in Toronto were immediately transported to the processing lab at The Hospital for Sick Children. Samples collected in Kingston were immediately processed in the lab on site.

### PBMC and granulocyte isolation

PBMCs and granulocytes were isolated using Ficoll Paque Plus (17-1440-03, GE Healthcare, Mississauga, ON). Ficoll Paque Plus was pipetted into the bottom of the processing tube, below the blood, according to the manufacturer's protocol with minor alterations. Blood layers were separated by centrifugation at 514 RCF for 25 min with the brake off. PBMCs were collected from the buffy coat interphase and washed with phosphate buffered saline (PBS) (see Supplementary Protocol 1). Following PBMC isolation, RBCs and granulocytes were isolated from the Ficoll Paque separation. Red blood cells were lysed, and pelleted granulocytes were washed in PBS. Concentrations of PBMCs and granulocytes were determined using a hemocytometer. Cells were resuspended in 10% dimethyl sulfoxide (DMSO) (Caledon, 4100-1-05) human AB (hAB) (VWR, CA45001-062) serum freezing media and frozen with 1 mL aliquots of 5–10 × 10^6^ cells/mL per vial. PBMCs and granulocytes were frozen at − 80 °C using a cell freezer for storage^37^.

### Biospecimen banking

Samples were stored locally and shipped to the POND McMaster Biobank (Hamilton, ON). Documentation of the biospecimen collection and aliquots was submitted electronically and in paper format with each shipment. Samples were stored in the POND McMaster Biobank at St. Joseph's Healthcare in Hamilton, ON. Biobank sample IDs were assigned to individual blood sample aliquots, and an up-to-date inventory of samples is maintained. Upon research proposal approval from platform leads, POND associated labs and collaborators request samples to be sent for research purposes.

### Flow cytometry and PBMC viability measures

A two-step sample viability check was used for quality control. Single aliquots of PBMCs were thawed in warm RPMI 1640 medium (SLM-240-B, Sigma-Aldrich, Oakville, ON). In step one, live cells were counted on a hemocytometer using the Trypan Blue exclusion test^38^. Samples with less than or equal to 5 × 10^5^ cells measured using the Trypan Blue exclusion test were considered to fail quality control. Samples that failed the Trypan Blue exclusion test were not used in subsequent flow cytometry. Samples that passed the trypan blue exclusion test criteria proceeded to step two where they were washed with fluorescence-activated cell sorting (FACS) wash (0.5% (w/v) BSA, 5 mM EDTA (pH 7.4–7.6), for 500 mL 2.5 g BSA, 5 mL of 0.5 M EDTA) prior to staining. 5 × 10^5^ cells were stained with Zombie Red Fixable Viability Kit (423,109, Biolegend, Cedarlane, Burlington, ON) and fluorochrome-conjugated antibodies against CD45 (BV510), each diluted 1:100 in a final volume of 50 µL of FACS wash, for 30 min at room temperature. Cells were then fixed using 1-step Fix/Lyse Solution (1X) for 10 min. PBMCs were washed with FACS wash and filtered through 0.45 µm mesh to ensure single-cell suspension before analysis on an LSR II flow cytometer (Becton, Dickinson and Company, Mississauga, ON). Cells stained Zombie Red positive were considered dead cells, while Zombie Red negative cells were considered viable. Samples with a Zombie Red viability of 70% or less were considered to fail quality control.

### Statistical analysis

Statistical analyses were performed using GraphPad Prism version 8.4.3. Analyses were conducted to assess differences in responses to the Child Immune Questionnaire, and differences between samples that passed and failed quality control. Fisher's Exact Tests were used to compare responses to the Child Immune History Questionnaire between TD and NDD, and to compare quality control pass rate by site. The Shapiro–Wilk Goodness-of-fit test was applied to continuous variables to assess normality. If the data were normally distributed, then the parametric *t*-test or one-way ANOVA was used. If the data was not normally distributed, then the non-parametric Mann–Whitney U test was used. All tests were 2-tailed, and *p* < 0.05 was considered significant.

## Supplementary Information

Supplementary Information 1.

Supplementary Information 2.

Supplementary Information 3.

## Data Availability

All research data is available upon request from the corresponding author.
