# Supplementary material for: Assessment of a multisite standardized biospecimen collection protocol for immune phenotyping in neurodevelopmental disorders
Source: Sci Rep. 2023 Apr 28;13:6971. doi: 10.1038/s41598-023-33380-z (PMC10147654; doi:10.1038/s41598-023-33380-z)

Supplemental Figure 1. Absolute Count of Leukocytes from CBC Report.

The mean count (±SD) of lymphocytes, monocytes, neutrophils, eosinophils, and basophils of participants who passed and failed quality control (QC).

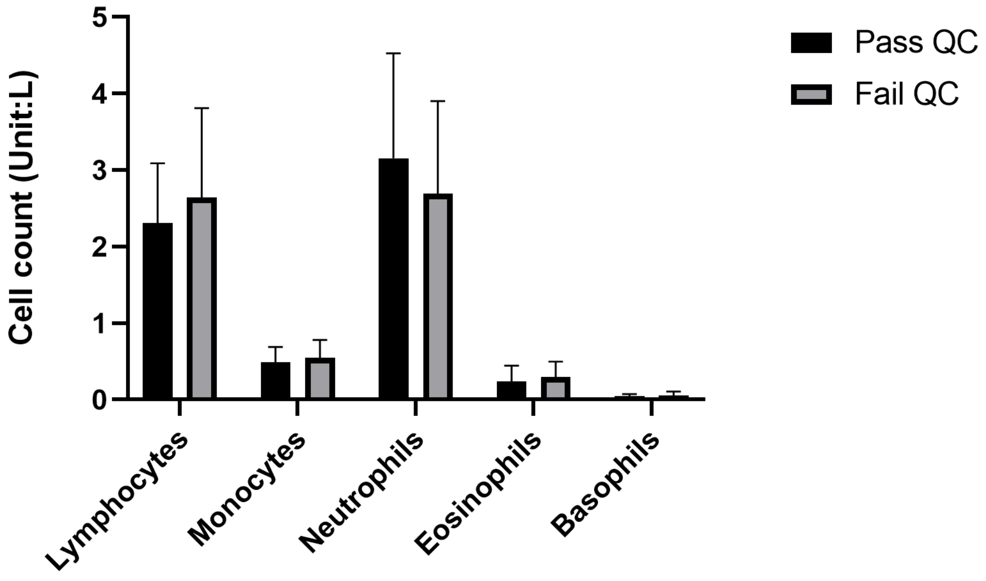

Supplement: Supplementary file 1 — Supplementary Information 1. [file 41598_2023_33380_MOESM1_ESM.pdf]
