# Supplementary material for: Assessment of a multisite standardized biospecimen collection protocol for immune phenotyping in neurodevelopmental disorders
Source: Sci Rep. 2023 Apr 28;13:6971. doi: 10.1038/s41598-023-33380-z (PMC10147654; doi:10.1038/s41598-023-33380-z)
Supplement: Supplementary file 2 — Supplementary Information 2. [file 41598_2023_33380_MOESM2_ESM.pdf]

POND- immunological questionnaire

CHILD IMMUNE HISTORY

Immunological Information:

Development of:

Allergy ☐ *Describe*: ____

Asthma ☐ Atopic dermatitis ☐ Allergic rhinitis ☐ Food allergy ☐

that was confirmed by physician and/or ancillary tests such as skin testing.

Number of days required oral (or intravenous) steroids at a dose of 1 or more mg/kg prednisone (or its equivalent) ____

Number of days of oral thrush in the first year of life: ______

Number of ear infections in last 3 years: _____ last 1 year: _____

Number of ear infections in last 3 years: _____ last 1 year: _____

Number of significant infections that required antibiotic treatment (e.g. pneumonia): ____

Adverse response to live vaccines, such as MMR and varicella zoster that required medical attention: Yes ☐ No ☐

If Yes, describe: ____

Describe outcome of chicken pox infection:

____

Has child had a case of autoimmune cytopenia (ITP, anemia)? Yes ☐ No ☐

Has child had a other autoimmune/rheumatological diseases? Yes ☐ No ☐

Describe_____________

Has child had required immune suppressive medications? Yes ☐ No ☐

Describe_____________

Has child experienced nail changes secondary to fungal infections? Yes ☐ No ☐

Has child had recurrent or difficult-to-treat skin warts? Yes ☐ No ☐

Is child growing and developing well (similar to siblings and peers)? Yes ☐ No ☐

Has child had cancer or tumor? Yes ☐ No ☐
