# Supplementary material for: Assessment of a multisite standardized biospecimen collection protocol for immune phenotyping in neurodevelopmental disorders
Source: Sci Rep. 2023 Apr 28;13:6971. doi: 10.1038/s41598-023-33380-z (PMC10147654; doi:10.1038/s41598-023-33380-z)
Supplement: Supplementary file 3 — Supplementary Information 3. [file 41598_2023_33380_MOESM3_ESM.pdf]

Standard Operating Procedure (SOP): Blood Processing and Storage

Date: April 2017
Approved by: Jane Foster, Dawn Bowdish
(McMaster BioBank Management)

1.0 PURPOSE

The purpose of this SOP is to outline the steps involved in isolating serum, plasma, peripheral blood mononuclear cells (PBMCs), and granulocytes from whole blood, as well as proper freezing procedures for banking of the samples

1.1 SCOPE

This SOP describes the necessary steps that need to be taken to ensure blood samples from participants are properly isolated and stored. Properly banked samples allows the immunophenotyping process to move forward. Blood products are a precious resource in the biobank and procedures must be followed to obtain products with high integrity and quality. This SOP does not cover detailed safety procedures for handling blood, thus personnel must follow institutional bio-safety guidelines.

1.2 MATERIALS AND EQUIPMENT

0.22µm Stericup Filter (Millipore Cat. # SCGPU01RE)
RPMI
hAB serum
PBS (warmed)
Ficoll
1X BD Pharm Lyse RBC lysis buffer
50mL Conical Tubes
Disposable Medical Examination Gloves
5mL, 10mL, and 25mL Pipets
Cell Freezer (Mr. Frosty)
Cryovials
Hemacytometer
Precision Pipettors (20µl and 1000µl)
Appropriate Sterile Pipette Tips

Blood Processing Binder
Waste Bag and Containers
Laboratory Coat
Centrifuge
Biosafety Cabinet (BSC)

1.3 PROCEDURE

A. PBMC Isolation and Storage

PBMC's come from the green top tubes. Step 3 and 4 below is a plasma separation step. Bank as described in plasma isolation protocol. Steps and Ficoll separation shown in Figure 1.

1. Preparation of hAB serum:
   a) First heat inactivate serum at 56°C in waterbath for 30 mins, swirling every 10 mins to reduce particulate formation.
   b) After heat inactivation, let cool and then filter through 0.2µm filter into a sterile tube in the BSC to remove any particulates that did form. Aliquot and Store AB serum at 4°C, or long term at -20°C.
   c) Prepare your 2X freezing media: 8ml hAB serum (80%) + 2ml DMSO 20% (any other 1:5 ratio is acceptable). Drip the DMSO into the hAB serum slowly while gently mixing (swirling motion).
   Note: DMSO clumps will disappear with sufficient gentle mixing. Do not pipet DMSO down side of polypropylene tube; clumps will form that will not dissolve
2. Chill Mr. Frosty in refrigerator for >30 minutes.
3. Centrifuge green top tubes for 10 minutes at 1500rpm (514 rcf) to separate plasma. *For further information, see Plasma Isolation.*
4. Remove yellow upper layer (plasma) from each green top tube. *Aliquot into cryovials (1mL/vial) for storage as described below.*
5. Using a 10mL pipet, transfer remaining blood sample into 50 mL tubes (maximum of 12.5 mLs / tube), record total volume of blood processed. *If there are multiple green top tubes from the same donor, these can be combined into the same 50mL tube, but only up to 12.5mLs*
6. Add an equal volume of PBS (up to 12.5mL maximum) to blood and mix gently with pipet. *PBS should be warmed at least to room temperature. Cold PBS may shock and damage the cells.*
7. Record information from tubes then dispose green top tubes in sharps container in BSC.
8. Slowly pipet equal volume of Ficoll under diluted blood so Ficoll layer forms below diluted blood layer (10ml blood + 10ml PBS + 20ml Ficoll). When done properly two (2) distinct layers should be observed.
9. Centrifuge for 25 minutes (**no brake)** at 1500rpm (514rcf) at **room temperature**.

10. A thin white interface should be observed between the top and middle layer after centrifugation. **NOTE:** This is the PBMC population to be isolated. *At this step, the separation is very sensitive. Take care not to jostle the tube.*
11. Remove the top layer of plasma using a 10mL pipette, leaving approximately 5mL above the interphase (be careful not to disturb the interphase). *Since you have already harvested the plasma above, this does not need to be kept. However, if you do keep this plasma layer, make note that it has been diluted with PBS and taken from this step in the protocol.*
12. Draw off the interphase (PBMC layer) with a 10mL pipette into another 50mL tube. Do not draw up too much of the Ficoll layer below, as this may result in granulocyte contamination. **Do not** combine interphase from multiple tubes; do a 1:1 transfer. ***Do not*** *discard the Ficoll and red blood cell layer, as the RBC layer contains the granulocytes.*
13. Dilute interface by pouring PBS into the tube up to 50mL (fill tube completely). Mix by inversion.
14. Centrifuge 10 minutes at 1500rpm (514rcf) at room temperature (brake on).
15. After centrifugation, pour supernatant into waste bucket and resuspend cells in 20mLs PBS using a pipetman. if pellet is small add 10 – 15 mLs PBS instead of 20 mLs. Or if pellet is large add 30-35mLs PBS instead of 20mLs.
16. Remove 10µl cell suspension and add equal amount of Trypan Blue in a microcentrifuge tube, and gently mix.
17. Using hemacytometer, count mononuclear cells (large, grayish cells), **not** RBCs (small, round, shiny cells). Calculate the number of cells/mL.
18. Centrifuge samples for 5 minutes at 1500rpm (514rcf) at room temperature.
19. Calculate the number of cyrovials required as 5 -10 x 10^6 cells/mL in each cryovial is desired. Label these vials before-hand, including the cell concentration.
    Example: 1x10^6 cells/mL in 20mLs= 20x10^6 cells total. If freezing at 5x10^6 cells/mL, with 1mL/ cryovial, resuspend in 4mL freezing media and distribute into 4 cryovials.
20. Resuspend the pellet in cold hAB serum and add an equal volume of freezing media by **dripping slowly** (20%DMSO + 80% AB serum) so that the final concentration is 5 – 10 x 10^6 cells / mL (i.e. 4 mLs total = 2 mL hAB + 2 mLs freezing media), gently mix well using a swirl motion. *Work quickly from this stage forward. DMSO is toxic to cells at room temperature. Transfer cells to the freezer as quickly as possible.*
21. Transfer 1 mL cell suspension into each labeled cryovial.
22. Store freezing container (Mr. Frosty) overnight at -80°C. Record the time of day the samples were put into the freezer. Transfer to long-term storage the next day.

Figure 1

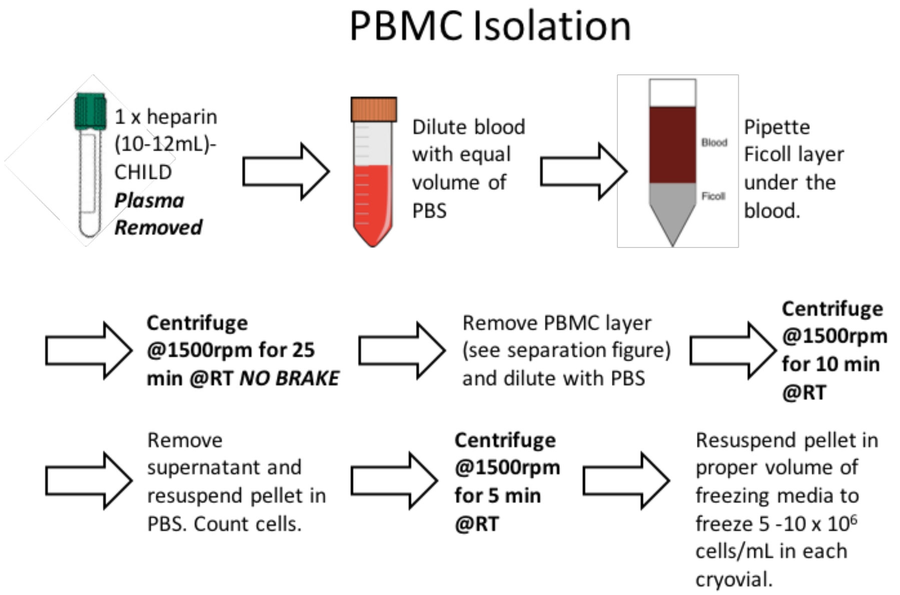

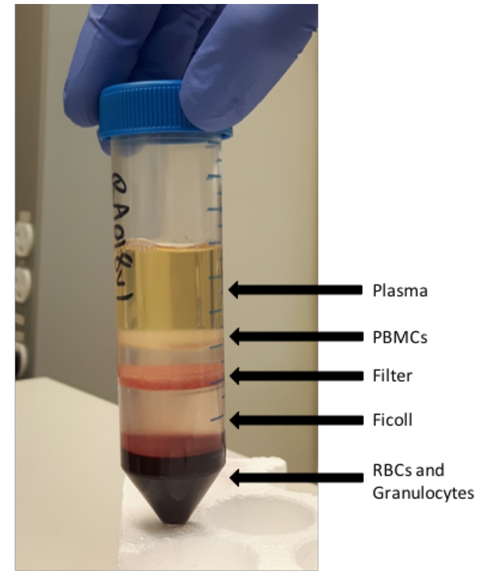

***Note:** This separation was done with a Leucosep tube. When done with a 50mL tube, the only difference is the filter will not be there. Everything else is the same.*

B. Granulocyte Isolation and Storage

Granulocytes come from green top tubes. This protocol starts after step 12 in the PBMC isolation protocol. Granulocytes are short-lived outside of the body, so they should not be left too long before they are banked. Steps shown in Figure 2.

1. Remove RBC and granulocyte layer from the separation tube into a 50mL tube, with 5mL maximum/tube.
2. Add 1X BD Pharm Lyse buffer to each 50mL tube at a 9:1 ratio to the RBC /granulocytes. Example: 5mLs of RBC/granulocytes, add 45mLs of lysis buffer. Mix by inversion or by gently vortexing.
3. Incubate at room temperature away from the light for 15 minutes.
4. Centrifuge at 200rcf for 5 minutes.
5. Remove supernatant without disturbing the pellet. *Steps 2-5 can be repeated, as once is not always enough to lyse all of the red blood cells.*
6. Add 2.0 ml 1X PBS.
7. Calculate the concentration of cells (cells/ml) using a hemacytometer. Record the cell concentration on the cryovials.
8. Centrifuge at 200rcf for 5 minutes.
9. Remove supernatant and resuspend in freezing media (same as step 20 above). *You will most likely only need one cryovial, as granulocyte recovery is relatively low.*
10. Store freezing container (Mr. Frosty) overnight at -80°C. Record the time of day the samples were put into the freezer. Transfer to long-term storage the next day.

Figure 2

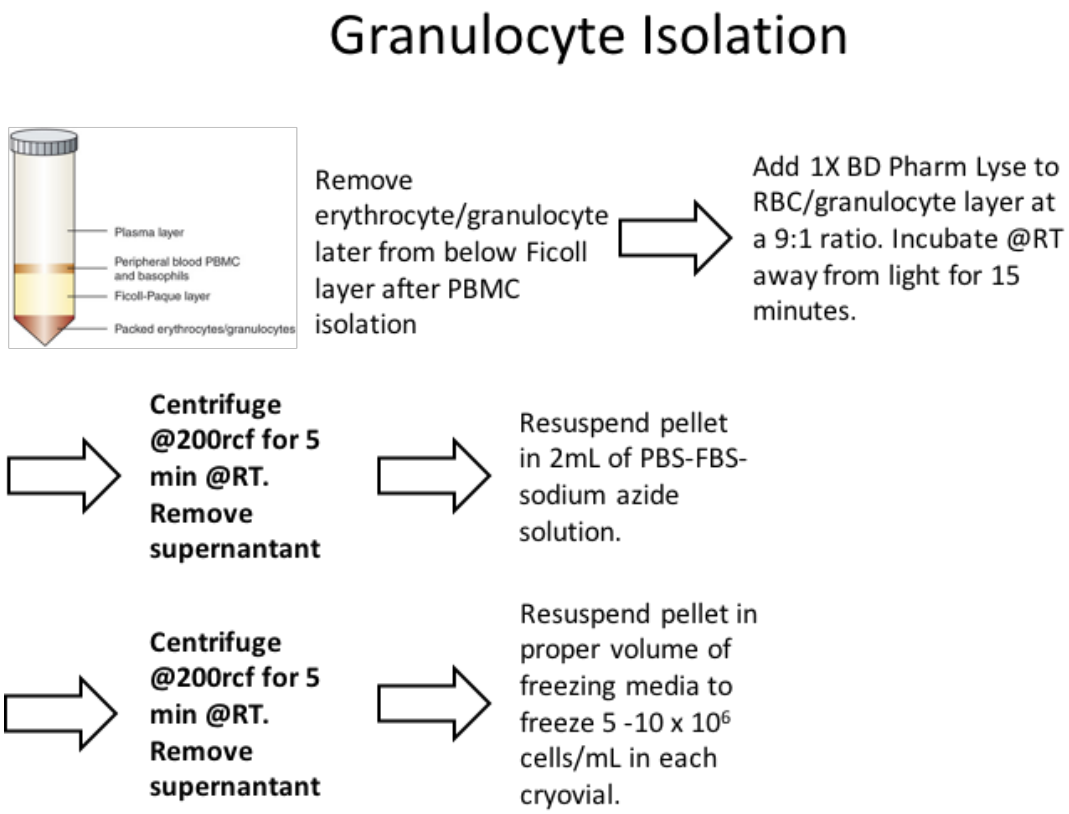

C. Serum Isolation

Serum comes from the red top blood collection tube. Samples can come in 6mL or 10mL blood collection tubes. Steps and separation shown in Figure 3.

1. After blood collection, place the red top tube upright at room temperature for 30 minutes. This will allow the blood to clot so the serum can be separated. *30 minutes is the standard time allotted for clotting. This must be done at room temperature directly after blood draw. If sample is to be shipped on ice, this step should be done prior to placing on ice. Once delivered, the sample should be warmed to room temperature and immediately continue with step 2.*
2. Centrifuge the clotted red top tube down at 1500rcf for 10 minutes at room temperature. *This will yield two distinct layers, one of straw coloured serum and another of red blood cells and all other cells and clotting factors. If needed, plasma isolation can be done in these conditions as well. So plasma and serum tubes can be centrifuged at the same time.*
3. Remove as much of the upper layer of serum as possible and aliquot 1mL into cryovials. *Be sure not to disturb the red blood cell layer. You can expect approximately 1-3 aliquots.*

Label cryovial with patient ID, birthdate, sex, date of processing, initials of the person who processed the sample, and what is in the tube (serum).

4. Store serum at -80°C. Record the time of day the samples were put into the freezer.

Figure 3

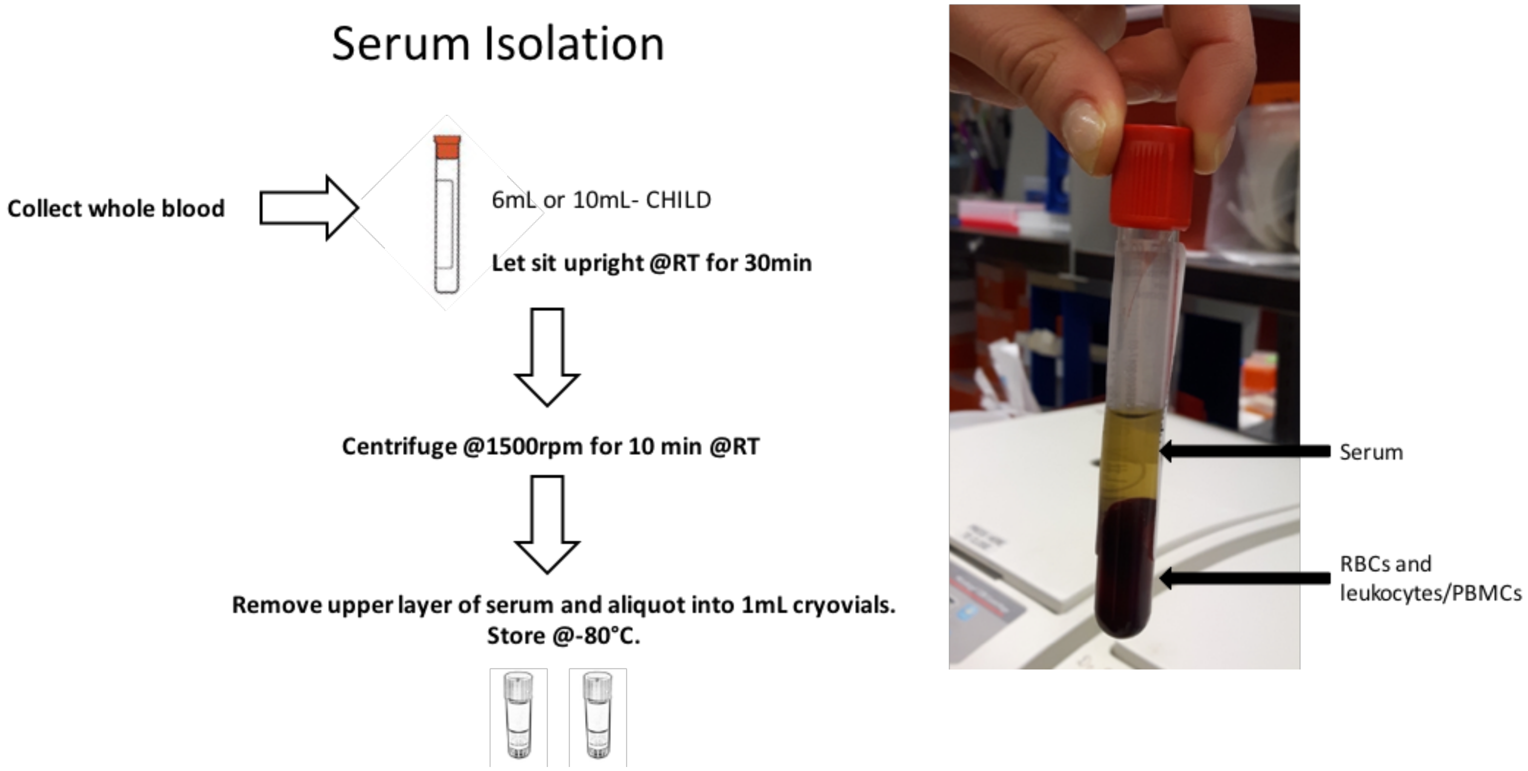

D. Plasma Isolation

Plasma comes from the green top tubes or the yellow top tubes. If something needs to be done the next day, it should be the isolation of the mother's plasma (yellow top). However, every effort should be made to accomplish this the same day. Steps and separation shown in Figure 4

1. Centrifuge the blood collection tube at 1500rpm (514 rcf) for 10 minutes at room temperature. *This will also yield two distinct layers. One of cloudy, straw coloured plasma, and another of red blood cells and all other cells.*
2. Remove as much of the upper layer of plasma as possible and aliquot 1mL into cryovials. *Be sure not to disturb the red blood cell layer. Labelling procedure same as step 3 of serum isolation. Mother's plasma labels should include mother's initials as well as the child's ID.*
3. Store plasma at -80°C. Record the time of day the samples were put into the freezer.

Figure 4

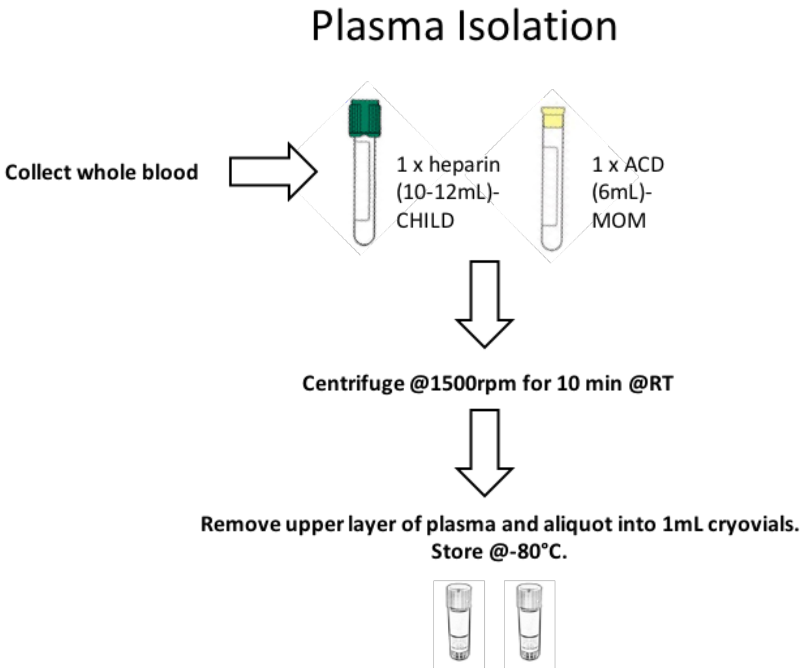
Plasma Isolation
Collect whole blood
1 x heparin (10-12mL)-CHILD
1 x ACD (6mL)-MOM
Centrifuge @1500rpm for 10 min @RT
Remove upper layer of plasma and aliquot into 1mL cryovials.
Store @-80°C.

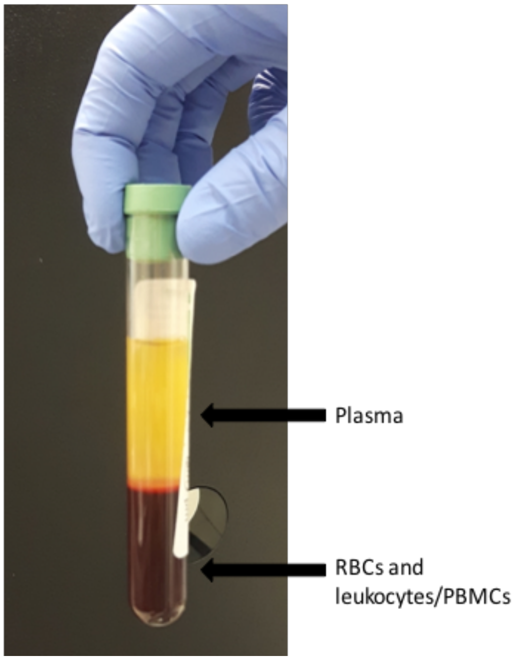
Plasma
RBCs and leukocytes/PBMCs
